# The Honey Bee Gene *Bee Antiviral Protein-1* Is a Taxonomically Restricted Antiviral Immune Gene

**DOI:** 10.3389/finsc.2021.749781

**Published:** 2021-10-20

**Authors:** Alexander J. McMenamin, Laura M. Brutscher, Katie F. Daughenbaugh, Michelle L. Flenniken

**Affiliations:** ^1^Department of Plant Sciences and Plant Pathology, Montana State University, Bozeman, MT, United States; ^2^Department of Microbiology and Immunology, Montana State University, Bozeman, MT, United States; ^3^Pollinator Health Center, Montana State University, Bozeman, MT, United States

**Keywords:** honey bee, antiviral, MF116383, *bee antiviral protein-1 (bap1)*, honey bee virus, honey bee immune system, ago2

## Abstract

Insects have evolved a wide range of strategies to combat invading pathogens, including viruses. Genes that encode proteins involved in immune responses often evolve under positive selection due to their co-evolution with pathogens. Insect antiviral defense includes the RNA interference (RNAi) mechanism, which is triggered by recognition of non-self, virally produced, double-stranded RNAs. Indeed, insect RNAi genes (e.g., *dicer* and *argonaute-2*) are under high selective pressure. Honey bees (*Apis mellifera*) are eusocial insects that respond to viral infections *via* both sequence specific RNAi and a non-sequence specific dsRNA triggered pathway, which is less well-characterized. A transcriptome-level study of virus-infected and/or dsRNA-treated honey bees revealed increased expression of a novel antiviral gene, GenBank: MF116383, and *in vivo* experiments confirmed its antiviral function. Due to *in silico* annotation and sequence similarity, MF116383 was originally annotated as a probable *cyclin-dependent serine/threonine-protein kinase*. In this study, we confirmed that MF116383 limits virus infection, and carried out further bioinformatic and phylogenetic analyses to better characterize this important gene—which we renamed *bee antiviral protein-1* (*bap1*). Phylogenetic analysis revealed that *bap1* is taxonomically restricted to Hymenoptera and *Blatella germanica* (the German cockroach) and that the majority of *bap1* amino acids are evolving under neutral selection. This is in-line with the results from structural prediction tools that indicate Bap1 is a highly disordered protein, which likely has relaxed structural constraints. Assessment of honey bee gene expression using a weighted gene correlation network analysis revealed that *bap1* expression was highly correlated with several immune genes—most notably *argonaute-2*. The coexpression of *bap1* and *argonaute-2* was confirmed in an independent dataset that accounted for the effect of virus abundance. Together, these data demonstrate that *bap1* is a taxonomically restricted, rapidly evolving antiviral immune gene. Future work will determine the role of *bap1* in limiting replication of other viruses and examine the signal cascade responsible for regulating the expression of *bap1* and other honey bee antiviral defense genes, including coexpressed *ago-2*, and determine whether the virus limiting function of *bap1* acts in parallel or in tandem with RNAi.

## Introduction

Conflict is the engine of evolution by natural selection and ecological diversification. Inter-organism conflict is most apparent when one organism parasitizes another, thus pitting the interests of two organisms against one another. Conflict between hosts and parasites drives the evolution of strategies to distinguish self from non-self and defend against invading pathogens, which are critical functions of immune systems (1). Metazoan immune systems are subdivided into the innate and adaptive responses which are further classified as humoral or cellular responses (1–6). Both humoral and cellular responses have been implicated in antiviral immunity in insects, including *Apis mellifera—*the western honey bee, a eusocial, cavity-nesting insect that is an important plant pollinator (7–9). The innate antibacterial and antifungal humoral responses in insects are induced by Pathogen Recognition Receptors (PRRs) which recognize Pathogen Associated Molecular Patterns (PAMPs) (10). The recognition of PAMPs by PRRs activates immune cascades—such as the Toll (NF-kB/Dorsal), Imd (NF-kB/Relish), and Jak/STAT pathways—which regulate the production and secretion of soluble antimicrobial peptides (AMPs) into the hemolymph [reviewed in (4–6, 10–13)]. These pathways are also involved in insect antiviral immunity, although the primary antiviral immune response is the RNA interference (RNAi) response [reviewed in (5)]. The RNAi response is a sequence-specific post-transcriptional gene silencing mechanism induced upon the recognition of double stranded RNAs (dsRNAs), including the replicative forms of single-stranded positive sense RNA viruses, by Dicer endoribonucleases. Dicer cleaves long dsRNAs into 21-22 bp short interfering RNAs, one strand of which (the guide strand) is retained by Argonaute-2 (Ago2), which is part of the RNAi-induced silencing complex (RISC). The RISC surveils the cell for complementary target RNAs, including viral genomes and transcripts, that are recognized, bound, and cleaved—which in turn limits virus infections (14–19) [reviewed in (4, 5, 20)].

Historically the invertebrate immune system was considered strictly innate (i.e., hosts respond naïvely to each encounter with a pathogen), while it was thought that the adaptive immune system, which includes pathway-specific and memory responses, was limited to jawed vertebrates [reviewed in (2, 11, 21)]. However, from an ecological and evolutionary perspective, it would be expected that insect hosts would have evolved immune systems with plasticity and specificity to respond to repeated exposure to pathogens within and across generations. Indeed, recent studies indicate that *Drosophila melanogaster* exhibits an adaptive immune response to viral infection (22–25). This response is predicated on the production of virus genome-derived DNA (vDNA) by reverse transcriptase, followed by vDNA transcription, and cleavage of the RNA transcripts into short interfering RNAs (siRNAs) (23, 24). Because RNAi proteins, including *dicer* and *ago2*, are involved in the conflict between hosts and viruses one would hypothesize that they are under positive selection and evolving rapidly (26, 27). In fact, analysis of dN/dS ratios of RNAi genes across multiple insect/arthropods indicate that these proteins have higher rates of adaptive evolution and selective sweeps compared to paralogous “housekeeping” genes, including in fruit flies and honey bees (28, 29).

Similar to other insects, RNA interference is antiviral in honey bees, as co-administration of a virus with virus-sequence specific dsRNA results in a reduction of virus abundance (30–37). For example, larval and adult honey bees fed diets with deformed wing virus (DWV) and DWV-specific dsRNA had greater longevity and lower DWV levels than bees exposed to DWV alone (32). In addition to sequence-specific RNAi, honey bees and bumble bees mount a virus-limiting response when exposed to dsRNA of any sequence (33, 36, 38–40). In contrast, this non-sequence specific dsRNA-triggered immune response has not been observed in highly investigated, solitary insects, including fruit-flies and mosquitos. Although the details of this non-sequence specific dsRNA-triggered antiviral mechanism have yet to be fully elucidated, the transcriptional level response to dsRNA both independently and in the context of virus infection have been well-characterized in honey bees, and the expression of select genes, including those involved in RNAi have also been examined in bumble bees (33, 36, 39–44). Specifically, a honey bee transcriptome sequencing study identified hundreds of genes differentially expressed in response to virus infection and/or dsRNA injection (36). The antiviral roles of two of the genes that exhibited greater expression in response to virus and/or dsRNA, *dicer-like* and the gene encoded by GenBank: MF116383, herein renamed *bee antiviral protein-1* (*bap1*), were confirmed *in vivo*. Specifically, RNAi-mediated reduction of expression of either of these genes in honey bees resulted in greater levels of the model virus Sindbis virus (SINV) compared to virus levels in bees treated with a non-specific dsRNA control (36).

As described above, Dicer is an endoribonuclease that processes dsRNA into siRNAs that are bound by Ago2/RISC and serve as sequence-specific guides for the targeted cleavage of cognate RNAs, including viral genomes and transcripts. The precise antiviral function of *bee antiviral protein-1* (*bap1*) (GenBank: MF116383.1) is still unknown. It was originally described as putative cyclin-dependent serine/threonine kinase based on *in silico* analysis (36, 45). It was one of the most highly differentially expressed genes in response to virus-infection (36). Therefore, to further characterize the gene, the full-length cDNA was Sanger sequenced revealing that the transcript length was longer than predicted (i.e., 5,158 nucleotide) and shared 91% nucleotide identity with an *Apis cerana* probable cyclin-dependent serine/threonine-protein kinase transcript (XM_017051141.1) and therefore it was similarly described in a previous publication (36).

In this study, we replicated the finding that the gene encoded by GenBank: MF116383 restricts virus infection and further characterized this gene. Sequence analysis using HMMER and domain searches failed to identify a putative kinase domain, therefore we renamed this gene *bee antiviral protein-1* (*bap1*) to better reflect its function. Analysis of the phylogenetic distribution of *bap1* and its orthologs revealed that it is taxonomically restricted to Hymenopteran insects and the German cockroach (*Blatella germanica*). To evaluate the potential evolutionary selective pressures on this gene, we calculated the site-wise dN/dS ratios and determined that the high substitution rate indicated by Bayesian phylogenetic inference is likely indicative of neutral selection across the majority of the gene, although some sites are under purifying and positive selection. To identify the pathways and proteins with which *bap1* may be associated, we used a weighted gene correlation network analysis (WGCNA) to identify highly coexpressed genes. This analysis revealed that *bap1* is coexpressed with several immune genes, most notably *ago2*, which we confirmed in an independent dataset. Together these data demonstrate that *bap1* is an important honey bee antiviral defense gene that is taxonomically restricted to primarily social insects (i.e., members of Apidae, Formicoidea, and *Blatella germanica*).

## Results and Discussion

### Virus-Limiting Role of Honey Bee *bap1* (GenBank: MF116383) Confirmed

For experiments described herein and previous studies, we utilized Sindbis virus (SINV), which is a well-characterized model virus that has been extensively utilized to investigate antiviral defense mechanisms in a wide range of insects including fruit flies, mosquitos, and honey bees (22, 33, 36, 46, 47). Therefore, use of this virus in experiments facilitates comparison of immune responses in both natural mosquito hosts and non-native hosts (i.e., honey bee and fruit fly) that have not co-evolved with this virus. Additional advantages of using SINV for *in vivo* experiments include: lack of confounding infection with this virus, facile virus purification from cultured cells, quantification of virions *via* plaque assay, lack of viral encoded RNAi suppressor proteins, and the ability to generate virus from a cDNA cloned construct that includes a carboxy-terminal green fluorescent protein (GFP) tag that facilities virus tracking *via* microscopy and assessment of virus abundance at the protein level *via* Western blot analyses (33, 36). A disadvantage of SINV is that it does not naturally infect honey bees. However, the generation of infectious clones of honey bee viruses is relatively recent and they have not been utilized for *in vivo* studies in laboratories beyond those in which they were developed (48–50). While some studies utilize honey bee virus preparations obtained from pupae, and while there are advantages of these lines of investigation, these virus preparations may include co-purifying viruses, as well as other proteins, and they are not a standardized source of infectious material. It is important to investigate host-virus specific interactions using a panel of viruses, including both model viruses and naturally infecting honey bee viruses, however this was beyond the scope of this study.

**Figure 1:**
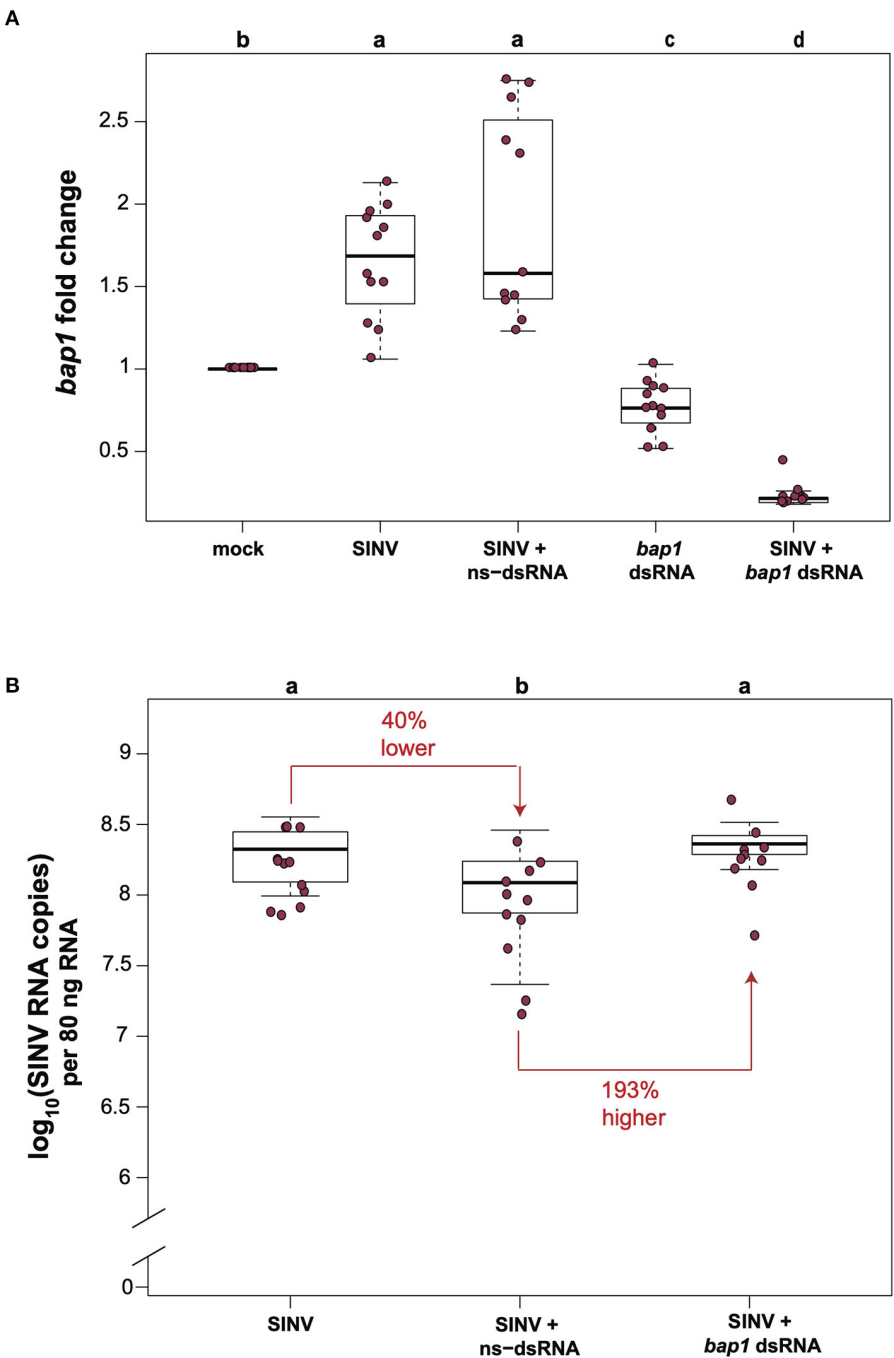
*Bee antiviral protein-1 (bap1)* is an antiviral immune gene. **(A)** Relative expression of *bap1* was assessed by qPCR, normalized to *rpl8* and fold changes were calculated relative to buffer-injected bees (mock). Bees infected with Sindbis-GFP (SINV) exhibited 1.65× greater expression of *bap1* compared to mock-infected bees (Dunnett's test, *p* < 0.001). Co-injection of virus and ns-dsRNA also resulted in a 1.92× increase of *bap1* expression at 72 h post-infection (hpi) compared to levels in mock-infected honey bees. RNAi-mediated gene knock-down was achieved by injecting *bap1*-seqeunce specific dsRNA which resulted in a mean 0.77× reduction, and a 0.23 fold reduction in *bap1* expression in bees co-injected with *bap1* dsRNA and virus relative to mock (*p* < 0.001). **(B)** Virus abundance was measured by determining the viral RNA copies using qPCR. Bees coinjected with ns-dsRNA and SINV and 40% lower SINV levels compared to SINV-only injected (*p* = 0.045). Bees with reduced *bap1* expression had 193% higher SINV levels relative to bees coinjected with SINV and ns-dsRNA (*p* = 0.016). Different letters above the treatments groups indicates a statistically significant difference.

In honey bees, dsRNA triggers both sequence specific and non-sequence specific virus limiting mechanisms that reduce virus abundance (30–36, 51). Therefore, the most relevant control for *in vivo* honey bee experiments aimed at investigating the impact of RNAi-mediated reduction of target gene expression on viral abundance approach, is a virus-infected and non-sequence specific dsRNA treated group, rather than comparison of virus and target gene expression levels in bees that did not receive dsRNA. To confirm that *bap1* limits virus infection, we carried out honey bee virus infection studies in the context of either non-sequence specific control dsRNA (ns-dsRNA) or *bap1*-specific dsRNA and quantified the relative expression of *bap1* and virus abundance using quantitative PCR (qPCR). Bees were collected for analysis at 72 h post-infection because previous work reported that RNAi-mediated knock down efficiency of *bap1* and the effect on virus abundance was most apparent at this time point (36). In this study, similar to a previous report, honey bees infected with the model virus Sindbis-GFP (SINV) exhibited 1.65× greater expression of *bap1* compared to mock-infected bees (Dunnett's test, *p* < 0.001) (Figure 1A) (36). Co-administration of virus and ns-dsRNA, which shares no homology with either virus or host sequences and is a viral-associated molecular pattern (VAMP), also resulted in a 1.92× increase of *bap1* expression at 72 h post-infection (hpi) compared to levels in mock-infected honey bees (Figure 1A). The RNAi-mediated reduction of *bap1* expression was achieved by injecting *bap1*-seqeunce specific dsRNA, which reduced mean *bap1* expression to 0.77× and 0.23×, relative to mock-infected bees, in honey bees injected with *bap1* dsRNA or with *bap1* dsRNA and SINV, respectively (Dunnett's test, *p* < 0.001) (Figure 1A).

Virus abundance in virus-infected bees compared to virus-infected bees co-injected with either ns-dsRNA or *bap1*-dsRNA was examined by determining the viral RNA copies (which represent both viral genomes and transcripts during active infections) using qPCR. In this study, similar to previous studies, co-injection of ns-dsRNA reduced SINV levels by 40% relative to levels in virus-infected bees (*p* = 0.045), a result which confirmed the virus-limiting impact of administration of immune system stimulating ns-dsRNA (Figure 1B). Furthermore, honey bees with reduced levels of *bap1* expression harbored 193% more virus than control bees which received ns-dsRNA (*p* = 0.016; Figure 1B). Virus abundance was similar in SINV-infected honey bees and SINV-infected bees that were co-injected with *bap1*-dsRNA, as compared to virus-only infected bees, due to the virus limiting impact of dsRNA (36). Therefore, the role of *bap1 in vivo* is best assessed by comparing virus abundance in the SINV-infected and ns-dsRNA treated bees vs. virus abundance in virus-infected bees treated with *bap1*-specific dsRNA (Figure 1B) Together, these results indicate that antiviral defense was hindered in honey bees with reduced *bap1* expression and confirm the findings of Brutscher et al. that *bap1* has an antiviral function in honey bees (36).

### Phylogenetic Analyses of *bee antiviral protein-1* Reveal It Is a Taxonomically Restricted, Divergent Gene That Is Evolving Under Neutral Selection

*Bee antiviral protein-1* (GenBank: MF116383) is a recently described honey bee antiviral gene transcribed from LOC725387 to produce a 5,158 nucleotide (nt) long transcript that produces a 1,511 amino acid protein (Supplementary Figure S1) (36). Although, transcriptome and *in vivo* studies indicate it is important for honey bee antiviral defense, the mechanistic and functional roles of *bap1* have not yet been elucidated. Therefore, we utilized HHpred with default parameters to identify any putative conserved amino acid domains to infer Bap1 function at the cellular level (52, 53). Hidden Markov Model searches against the protein data bank (PBD), Pfam-A_34, Swissprot, COG_KOG, or NCBI Conserved Domains databases all failed to identify any conserved domains. Therefore, in contrast to initial reports based on automated annotations, the gene encoded by GenBank: MF116383 does not likely have a kinase domain, and therefore *bap1* is a more accurate name for this gene and associated protein (ASQ15625).

**Figure 2:**
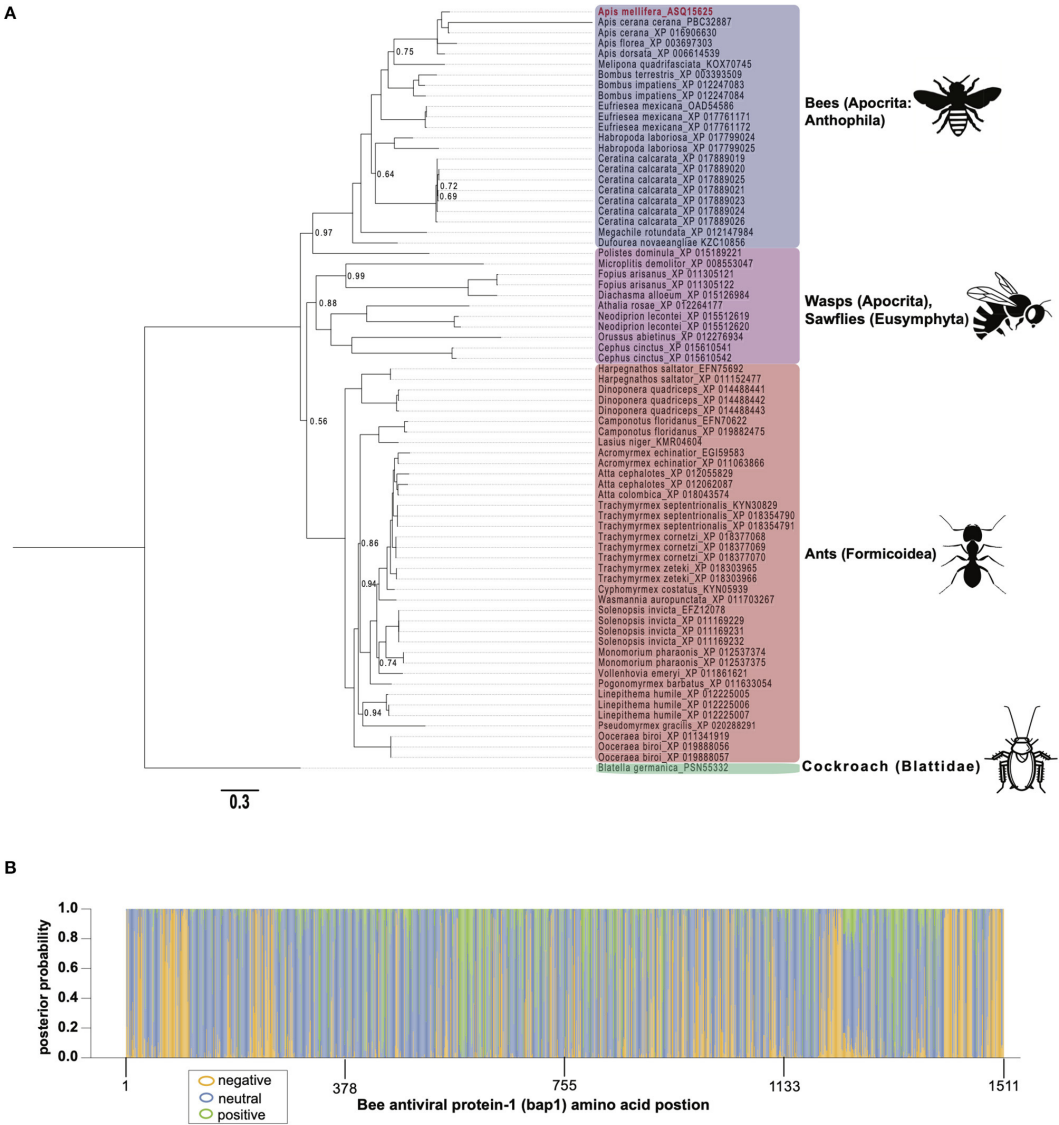
Bee antiviral protein-1 phylogenic relationship to other Hymenopteran orthologs inferred from amino acid sequence. **(A)** Majority rule Bayesian consensus tree of Bap1 homologs derived from Bayesian analysis of amino acid alignment implemented in Mr. Bayes v3.2 using a Jones substitution model. Numbers on branches and nodes are posterior probabilities (0-1), though posterior probabilities values of 1 are not shown to improve clarity. The scale bar corresponds to proportion of amino acid change. Accession numbers are included on the branch tips and in Supplementary Table S2. **(B)** A corresponding majority Rule Bayesian consensus tree derived from Bayesian analysis of a codon alignment was used in a selection analysis in the CODEML package in Phylogenetic Analysis by Maximum Likelihood (PAML) 4.9. The Bayes Empirical Bayes method under model 2, which assumes 3 site classes (negative, neutral, and positive selection), was used to calculate the posterior probability that each amino acid position belonged to each site class. The posterior probabilities were then plotted as a stacked bar chart along the length of Bap1 amino acids. This method shows clear diffuse neutral selection with regions under strong positive or negative selection.

To gain insight on the phylogenetic distribution of *bap1*, which may also provide functional insights, we identified orthologous proteins in other insects and assessed their phylogenic relationship (Figure 2A). Honey bees belong to the order Hymenoptera, which is a large order of insects with over 150,000 species. Orthologs of *bap1* were identified in the major lineages of Hymenoptera, including Apocrita (wasps and Anthophila), and Eusymphyta (which include sawflies, horntails, and wood wasps). Whereas, orthologous genes were not identified in Diptera (including *Drosophila melanogaster*, as well as mosquitos in genera *Culex, Aedes*, and *Anopheles*) or most other insect sequences in NCBI databases with the exception of one protein in *Blatella germanica* (the German cockroach).

Orthologs of *bap1* were identified *via* the Position-Specific Iterated Basic Local Alignment (PSI-BLAST) tool on the website of the National Center for Biotechnology Information (NCBI) (Supplementary Table S2) (54–56). We identified 73 orthologs with percent amino acid identities ranging from 17 to 82% (Supplementary Figure S1; Supplementary Table S3). The protein sequences were aligned using MEGA X (MUSCLE with default parameters) (57) and analyzed by Bayesian inference phylogenetic analysis using MrBayes v3.2.7a (58, 59) with *Blatella germanica* as the out group since it is the only non-Hymenopteran taxon on the tree (Figure 2A). Unfortunately, Euymphyta and Apocrita (aside from Anthophila) remain very under sampled on this tree due to a lack of genome assemblies or low-quality genome assemblies. It is interesting that *bap1* orthologs were not identified in model insects such as *Drosophila melanogaster* (Diptera)*, Spodoptera frugiperda* (Lepidoptera), or *Tribolium castaneum* (Coleoptera). Phylogenomic analysis using 1,478 ortholog groups and published transcriptome data sets suggests the ancestors of Hymenoptera and Blattodea split roughly 400 million years ago, compared to the ancestors of Diptera, Lepidoptera, and Coleoptera, which are phylogenetically more similar to Hymenoptera, having diverged about 350 million years ago (60). Even though bees and cockroaches diverged long before bees and flies, a *bap1* ortholog was identified in *Blatella germanica*, the German cockroach. Interestingly, experiments aimed at investigating gene function in this species using RNAi-mediated gene knockdown, revealed that *dicer2* expression was increased in response to ns-dsRNA (61). Although complete transcriptome profiling of dsRNA-treated cockroaches has yet to be evaluated, we hypothesize that *bap1* orthologs were retained in several phylogenetic lineages that have a transcriptional response to dsRNA, and that this response may be antiviral– as it is in honey bees and bumble bees. Although, this hypothesis remains to be tested. In line with our hypothesis, fruit flies and mosquitos, which do not have *bap1* orthologs, also do not exhibit virus-limiting responses to ns-dsRNA. However, there are organisms that limit virus infection in response to ns-dsRNA that lack a *bap1* ortholog (e.g., the sand fly, *Lutzomyia longipalpis*) (62). It not clear whether this phenotype in *L. longipalpis* is a retained (from an ancestor) or derived phenotype.

To confirm that some insects lack a *bap1* ortholog, PSI-BLAST searches directed to several genomes (i.e., *Drosophila melanogaster, Lutzomyia longipalpis*, and *Aedes albopictus*) were performed, and a Hidden Markov Model approach to identify deep homologs (JackHMMER) was utilized; all failed to identify additional orthologs (63). This indicates that *bap1* is a taxonomically restricted gene either due to lineage-specific losses or high divergence (i.e., high average amino acid substitution at each position) in other lineages thereby making orthologs unidentifiable.

The phylogenetic relationships based on the Bap1 protein sequence, grouped wasps and sawflies together, which was likely an artifact of long branch lengths, and not an accurate representation of lineage (Figure 2A). If the Bap1 protein tree reflected the true phylogenetic relationships within Hymenoptera, you would expect Eusymphyta to form the basal clade of Hymenoptera (64). Instead, in this tree, Anthophila appears to have split from the rest of Apocrita and Formicoidea before they diverged, when in fact Anthophila is the most derived clade within Hymenoptera (64).

The high rate of amino acid substitution in Bap1 orthologs (0.3 amino acid substitutions per position) is indicative of rapid evolution (Figure 2A). To contextualize the seemingly high amino acid substitution rate on the Bap1 tree (Figure 2A), a parallel analysis was carried out on RPB1, the largest subunit of eukaryotic DNA-directed RNA polymerase II, which showed a more than 10-fold lower substitution rate (0.02, Supplementary Figure S2; Supplementary Table S4) (65). Given the high substitution rate in Bap1 we hypothesized that Bap1 was under positive selection causing divergence between lineages. A gene is under positive selection when the ratio of non-synonymous mutations (a change in amino acid; dN) to synonymous mutations (no amino acid change, dS) as >1. This indicates some selective pressure is driving the fixation of advantageous mutations in lineages. A gene is under negative selection when the dN/dS ratio is <1, which indicates selection pressure is purging amino acid-changing mutations because they are deleterious. One might expect that an antiviral gene is under positive selection due to the arms-race between a pathogen an its host, which may favor non-synonymous mutations (26). Alternatively, genes that are important to numerous important biological processes, including some immune genes, could be under negative selection, in order to ensure structural and functional preservation. Furthermore, individual amino acids can experience unique selective pressure and, therefore, whole-gene dN/dS ratios are unlikely to be reflective of evolution at individual amino acids. A maximum likelihood analysis of site-wise dN/dS ratios using PAML revealed that *bap1* has codons evolving under negative (260 sites, dN/dS = 0.16) and positive selection (123 sites, dN/dS = 1.94) but most sites are evolving under neutral selection (797 sites, dN/dS = 1.00) (Figure 2B; Table 1; Supplementary Figure S3; Supplementary Tables S5, S6, S10) (66). This is in stark contrast to RPB1, the largest subunit of eukaryotic DNA-directed RNA polymerase II, which also has sites under neutral (191 sites, dN/dS = 1) and positive (98 sites, dN/dS= 14) selection, but most sites are under very strong negative selection (1,314 sites, dN/dS = 0.0006), which is characteristic of highly conserved proteins involved in fundamental biological processes, including transcription (Supplementary Figures S2B, S4; Supplementary Tables S7–S10). An Empirical Bayes approach was used to calculate the posterior probability that each amino acid was evolving under negative, neutral, or positive selection (Figure 2B; Supplementary Figure S2B). Overall, the pattern of selection and phylogenetic distribution indicate that the *bap1* is a taxonomically restricted ortholog group evolving primarily under neutral selection causing rapid divergence, as compared to a control gene (RPB1) (Figure 2; Supplementary Figure S2).

**Table 1:** Bee antiviral protein-1 (*bap1*) orthologs are primarily under neutral selection.

	**M0**	**M1**	**M2**
Description	Assumes one dN/dS ratio for whole gene	Assumes near-neutral selection	Assumes 3 classes with varying selection (negative, neutral or positive)
-lnL	−160,366.55	−154,730.907	−154,513.0653
Test Statistic (null is M0)		11,271.286	M2-M0: 11,706.97
			M2-M1: 434
*p*-value		<1 × 10^−15^	M2-M0: <1 × 10^−15^
			M2-M1: *p* < 0.001
dN/dS	0.628	negative = 0.146, neutral = 1.000	negative = 0.16
			neutral = 1.00
			positive = 1.94

*Measures of selection (dN/dS) were calculated for bap1 (GenBank: MF116383) orthologs under three models. M0 assumes one dN/dS ratio for the whole protein. M1 assumes the protein is evolving under near-neutral selection and calculates dN/dS for two site classes (dN/dS = 1 and dN/dS <1). Lastly, M2 assumes there are three codon site classes in the protein (dN/dS < 1, dN/dS = 1 and dN/dS >1). The tests show M2 is the best model and that bap1 orthologs have sites under negative, neutral, and positive selection*.

### *Bee antiviral protein-1* Is a Disordered Protein That Is Trafficked to the Endoplasmic Reticulum

**Figure 3:**
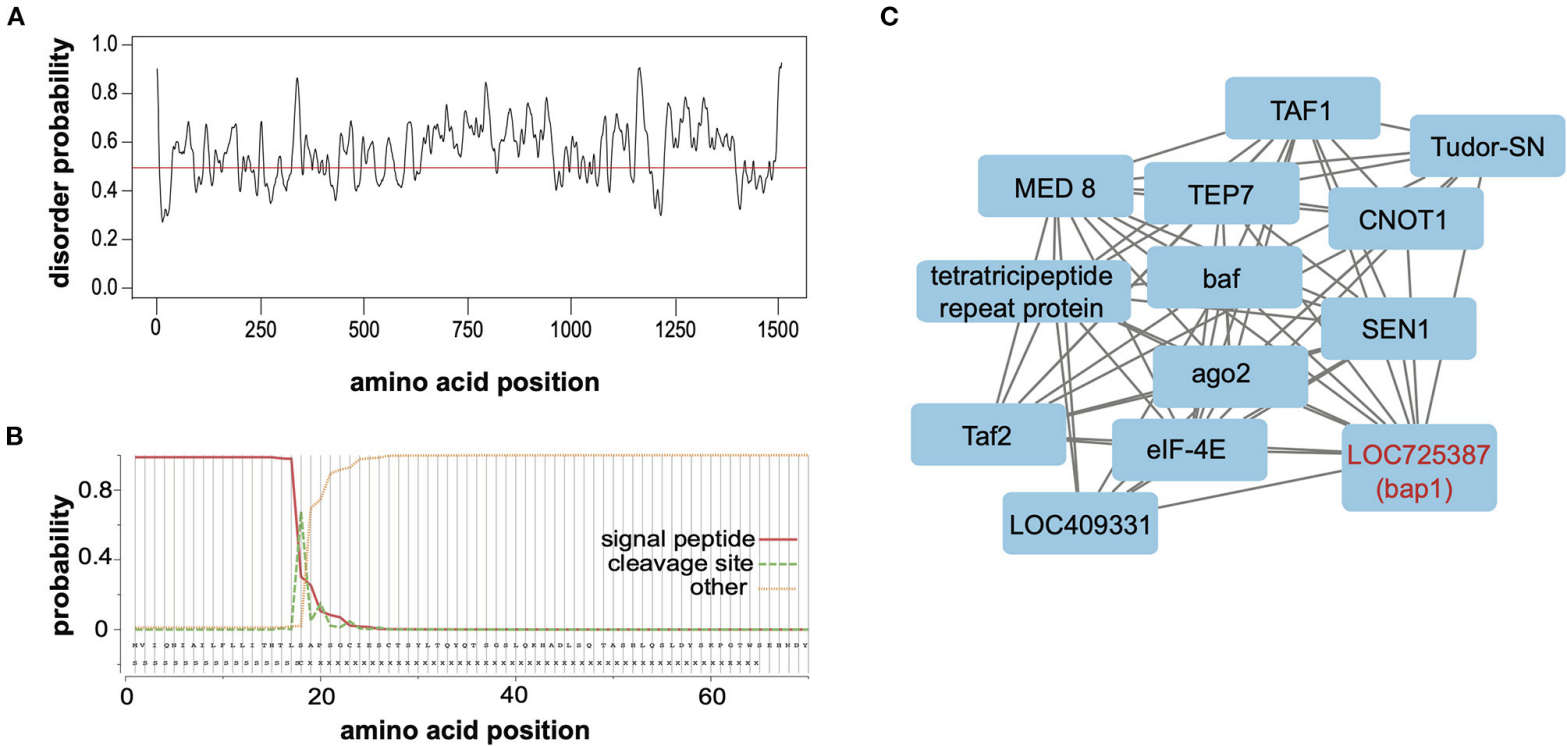
Bee antiviral protein-1 (Bap1) is a disordered protein coexpressed with several other immune genes. **(A)** The PrDOS web tool was used to predict disorder probability at each amino acid position in Bap1. PrDOS predicted that 67.2% of Bap1 has a more than 50% probability of being disordered. **(B)** SignalP-5.0 was used to predict the presence of a signal peptide on the N-terminus. It is highly likely that bap1 has a signal peptide (probability of 0.983) that is cleaved off between amino acids 21 and 22. **(C)** Weighted gene correlation network analysis (WGCNA) was used to identify genes that are highly coexpressed in various contexts (i.e., virus infection or dsRNA administration). WGCNA identified that module (a highly coexpressed cluster of genes) #38 was associated with virus infection with a correlation of 0.54 (*p* = 1 × 10^−4^, Supplementary Figure S5) and contained *bap1*. This module also contained several immune genes including *ago1, Tudor-SN*, and *TEP7*. For full subnetwork see Supplementary Figure S6.

Relaxed evolution is a hallmark of disordered proteins due to relaxed constraints on their structure (67–69). To ascertain whether the largely neutral selection on *bap1* orthologs (Figure 2B) was due to relaxed structural constraints, PrDOS (protein disorder prediction system) was used to calculate disorder prediction at each amino acid position with the default 5% false positive rate (Figure 3A) (70). This analysis indicated that the majority (i.e., 67.2%) of the Bap1 amino acid sequence is disordered or lacks structure (i.e., above 0.5 probability, Figure 3A). Intrinsic disorder in protein structure allows the function of a protein to be highly modular since it allows the binding of multiple partners, including proteins and nucleic acids (71). In fact the relaxed structural constraint, leading to decreased packing density, is associated with more rapid evolution (higher substitution rates) of disordered proteins, which is in-line with the bioinformatic analyses of *bap1*, described herein (Figure 2A) (67–69, 72). Due to their modularity, intrinsically disordered proteins (IDPs) often act as hubs in protein interaction networks (73–75). When bound to their target, many IDPs adopt a more structured conformation (76, 77). Future analyses, including the use of nuclear magnetic resonance (NMR) and X-ray scattering techniques, are required to definitively characterize the structure of the Bap1 protein in bound or unbound states.

To examine the putative subcellular localization of the protein encoded by *bap1*, we used SignalP-5.0 to predict the presence of a signal peptide on the N-terminus (78). Signal-P predicted the presence of a signal peptide with a probability of 0.983 with a cleavage site between amino acids 21 and 22 (Figure 3B). Signal peptides are N-terminal sequences on proteins that promotes targeting to the endoplasmic reticulum (ER), *via* recognition by the signal recognition particle (79, 80). Once translocated across the membrane into the ER lumen, the protein can either be released from the membrane *via* cleavage of the signal peptide by signal peptidases or it can remain associated with the membrane (80–82). This process can happen co-translationally for larger proteins or post-translationally for smaller proteins (83). Once in the ER, however, the protein may be secreted, stay in the ER, or be sorted into intracellular compartments like the Golgi, endosomes or lysosomes (80, 84, 85). We were unable to identify any further localization signals that might indicate subcellular localization. Therefore, our data indicate that Bap1 likely resides in a membrane-bound compartment (or may be secreted) and may serve as a hub of molecular interactions (i.e., nucleic acid-protein or protein-protein) in the honey bee antiviral response. Further work to identify how Bap1 is limiting virus infection would be an exciting avenue of research.

### *Bee antiviral protein-1* Is Co-expressed With Several Honey Bee Immune Genes

Genes with a coordinated function (e.g., act in antiviral defense) are often co-expressed. The honey bee antiviral gene *bap1* was first described and annotated in a transcriptome level study of honey bees infected with a model virus in the presence and absence of dsRNA species (36). This study assessed gene expression in individual bees over a time course (i.e., 6, 48, and 72 h post-injection) as compared to mock-infected (i.e., buffer-injected) control bees and presented fold-change and statistical significance data independently for each annotated gene at all time-points (36). To further evaluate the genes highly coexpressed with *bap1* we constructed a signed weighted gene correlation network from our previously published transcriptome dataset using the R package Weighted Gene Correlation Network Analysis (WGCNA) (36, 86, 87). After filtering, WGCNA identified 39 modules, which are clusters of highly co-expressed genes. Select modules are discussed below and pairwise correlations between genes in the identified modules are presented in Supplementary Tables S13–S20. There were three gene modules that most correlated with virus (SINV) infection (i.e., #8, #15, and #38), and one module that best correlated with dsRNA treatment (i.e., #20) (Supplementary Figure S5). These modules are described below, and the genes and gene-gene correlations of additional modules are included in Supplementary Tables S17–S20.

Gene module #8 and SINV infection had a correlation coefficient of 0.56 (*p* = 4 × 10^−5^) (Supplementary Figure S5). This is a small module with only 38 genes, therefore gene ontology analysis of this group was not possible, and most of the genes in this module have no known or predicted function. However, interestingly, several predicted non-coding RNAs (ncRNAs) belong to this module (e.g., LOC100578712, LOC102654940, LOC102655797, and mir3764) (Supplementary Table S13). Non-coding RNAs perform a wide range of functions, but most notably pre- and post-transcriptional regulation of gene expression through methylation or RNAi, respectively (88–91). Non-coding RNAs may have either a pro- or anti-viral role [reviewed in (92)]. One gene in this module was IGFn3-6 (also known as DSCAM2-like), a gene in the immunoglobulin-like superfamily. DSCAM2 is a gene that undergoes extensive mutually exclusive alternative splicing to create a wide range of somatic diversity. It is involved in neuronal development in *Drosophila melanogaster* and DSCAMs have also been implicated in immunity, including potential roles in antiviral immunity (93–96). In honey bees (*A. mellifera syriaca)* DSCAM2 SNPs were also associated with *Varroa destructor* mite resistance phenotypes, and DSCAM exhibited higher expression in virus-infected bees than in control bees (33, 97).

Gene module #15 and SINV infection had a correlation coefficient of 0.54 (*p* = 8 × 10^−5^) (Supplementary Figure S5). This module contains 122 genes, but since many of them lacked functional assignments, no significant gene ontology terms were detected (Supplementary Table S14). Like gene module #8, gene module #15 also included several non-coding RNAs (LOC100577428, LOC102655361, and LOC102654929), as well as genes involved in pathogen recognition (*pgrp-s2*), transcriptional repression (*snail*), cell morphogenesis (*trbl*), cell migration regulation (*PVF1*), and a gene involved in the anti-parasitoid response (*Flo2*). *Pgrp-s2* also exhibited increased expression in this data set in response to SINV infection. Pgrp-s2 is a member of a class of pathogen recognition receptors that activate the Toll or Imd pathway in response to pathogens, and it has also been implicated in antiviral immunity in *Bombyx mori* where it activates the Imd pathway (98). The Toll and Imd pathways, which are mediated by NF-kB-like transcription factors are both important for antiviral defense against certain viruses in *Drosophila melanogaster* and other insects (99–101) [reviewed in (5)]. The mechanism of activation of these pathways by virus infection is unclear and their precise role is not fully characterized, though Imd may contribute through regulation of apoptosis (101).

Gene module #38 and SINV infection had a correlation coefficient of 0.54 (*p* = 1 × 10^−4^, Supplementary Figure S5). This module contains 481 genes, similar to other modules, gene ontology could not be assessed since numerous genes in this cluster lacked functional annotation. However, this module included genes involved in metabolism, transcription, translation, and immunity (Supplementary Table S15). The immune genes in this module included *bap1* (LOC725387), which was co-expressed with additional immune genes *tep7, tudor-SN*, and *ago-2* (15, 16, 102–104) [reviewed in (5)]. Thioester containing protein 7 (TEP7) is a member of the thioester containing protein family which includes vertebrate complement proteins [reviewed in (105)]. In fruit flies and mosquitoes, *TEP* expression is likely regulated by JAK in response to bacterial infection and they function in bacterial recognition, opsonization and phagocytosis [reviewed in (105, 106)]. However, in *Aedes aegypti* TEP1 and TEP2 limit West Nile virus infection and TEP is indirectly involved in combatting Dengue virus (107, 108). Lastly, Tudor-SN is a nuclease and a core component of RISC of Drosophila that is also involved in cleavage of hyper-ADAR-edited dsRNAs (109–111). In summary, *bap1* is co-expressed with several genes that are implicated in antiviral immunity in several species (Figure 3C).

Gene module #20 and dsRNA-treatment were moderately associated with a correlation coefficient of 0.35 (*p* = 0.02) (Supplementary Figure S5; Supplementary Table S16). This module contained 806 genes and had significant enrichment for genes involved in nucleic acid binding (*p* = 0.016), metal ion binding (*p* = 0.016), and genes with a zinc finger C2H2-like domain (*p* = 0.002). These groups comprise proteins with a wide range of functions including protein, DNA, ssRNA, and dsRNA binding functions [reviewed in (112)], sometimes interacting with both nucleic acids and proteins. The zinc finger C2H2 domain is by far the most common domain in metazoan transcription factors [reviewed in (113)], but their wide range of binding activities suggests functional diversity. Indeed, there are antiviral mammalian zinc finger nucleases which can either directly bind to viral nucleic acids and promote their degradation, or otherwise regulate the immune response [reviewed in (114)].

### Confirmation of *bap1* and *ago2* Coexpression in Honey Bees

To confirm the co-expression of *bap1* and *ago2*, a *post-hoc* analysis was performed on a dataset, composed of previously published data and data generated in this study (Figure 1B) (47). The previously published dataset was from a study that determined that the heat shock response in honey bees is antiviral (47). Specifically, expression data from individual bees that were (a) SINV-infected, (b) exposed to heat shock (42°C for 4 h) only, or (c) both virus-infected and heat shocked (47). This combined dataset was utilized for *post-hoc* analysis because bees were subjected to various treatments that impact *bap1* expression, including heat shock which increases *bap1* expression (47). The association between *bap1* and *ago2* expression was analyzed using the following linear model:


$$\begin{array}{l} \text{ago}2\text{ fold change }\sim \;\beta_{0}+\beta_{1}*bap1\text{ fold change}\\ \;*\beta_{2}*\text{SINV RNA copies} \end{array}$$

**Figure 4:**
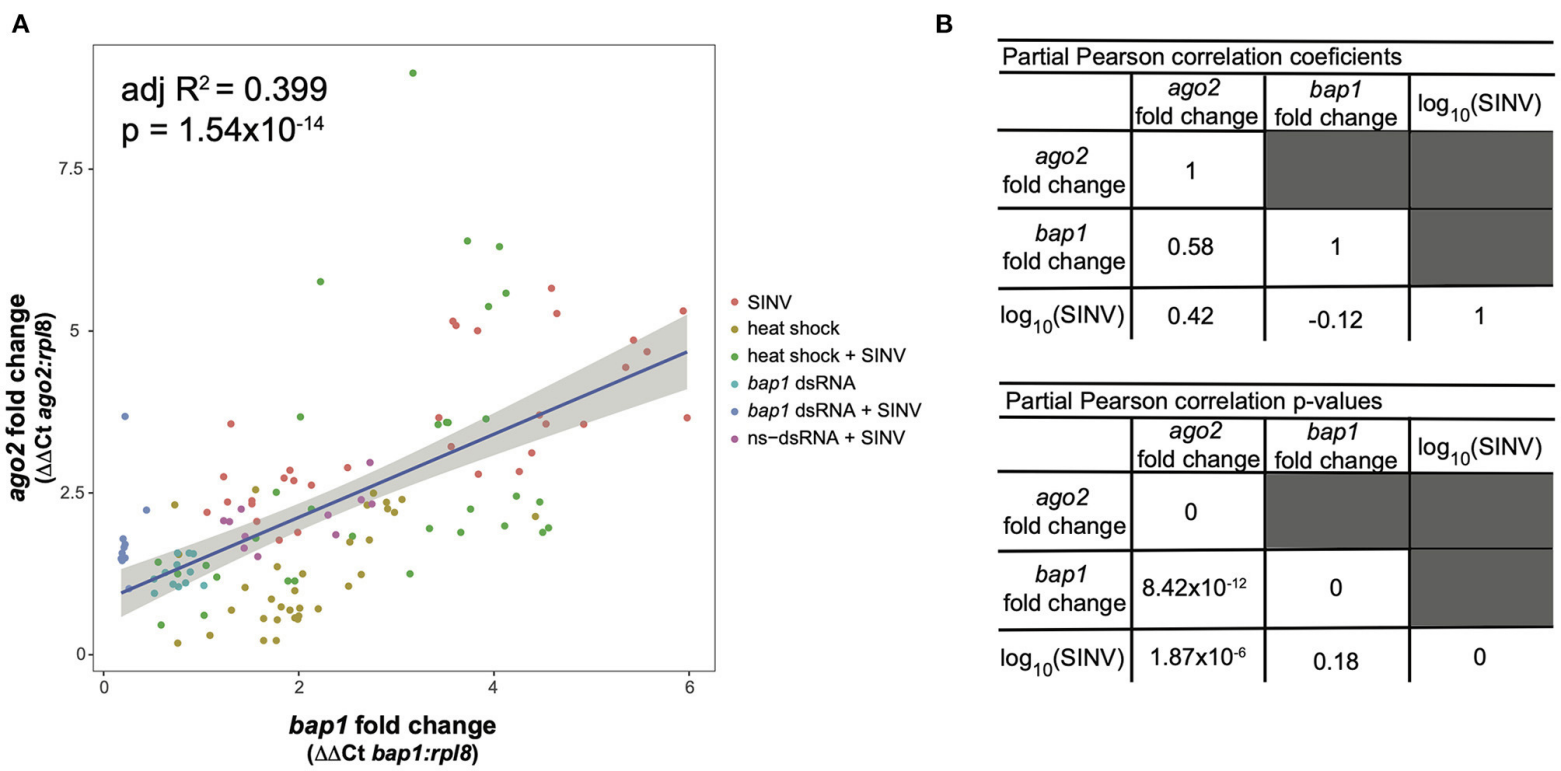
Expression of *bap1* and *ago2* is associated after accounting for the effect of virus abundance. The relative expression of *bap1* and *ago2* in bees that received a variety of treatments was assessed by qPCR relative to mock infected bees. **(A)** *Bap1* and *ago2* fold changes are associated in bees that received a variety of treatments (adjusted *R*^2^ = 0.399, *p* = 1.54 × 10^−14^). **(B)** A partial Pearson's correlation coefficient was calculated to test for a correlation between *bap1* and *ago2* expression after accounting for the effect of virus (SINV) abundance. While there is a correlation between *ago2* and SINV level (*r* = 0.42, *p* = 1.87 × 10^−6^) there was no correlation between *bap1* and SINV. After accounting for the effect of SINV on *ago2* expression, there is a strong correlation between *ago2* and *bap1* (*r* = 0.58, *p* = 8.42 × 10^−12^).

This model structure was selected because SINV and *ago2* expression are also weakly associated (adjusted *R*^2^ = 0.06, *p* = 0.003, log likelihood = −233.4), and therefore including the interaction term significantly improved the model. The model indicates that *bap1* expression (fold change relative to control buffer-injected bees) is a good predictor of *ago2* expression (Adjusted *R*^2^ = 0.399, *p* = 1.55 × 10^−14^, log likelihood = −203.3, Figure 4A). This model also indicates that SINV remains a weak predictor of *ago2* expression, which was expected since *ago2* expression is induced by SINV infection (36). However, the chosen model structure explains only 40% of the variance in the dataset, indicating there are additional variables, which are not accounted for in the model, that effect *ago2* expression. See Table 2 for full model output.

**Table 2:** Linear model output for the association between *bap1* and *ago2* expression.

**Term**	**Estimate**	**Standard error**	***t*-value**	***p*-value**
Intercept	0.444	0.25	1.77	0.079
*bap1* fold change	0.733	0.092	7.99	7.26 × 10^−13^
SINV RNA copies	3.92 × 10^−9^	1.39 × 10^−9^	2.82	0.0056
bap1*SINV	−7.23 × 10^−10^	3.28 × 10^−10^	−2.213	0.0287

*Ago2 expression was modeled as a function of bap1 expression coupled with SINV RNA copy number. The model indicates that, after accounting for the interaction between SINV RNA copies and bap1 expression, the expression of bap1 and ago2 are highly associated, and corroborated WGCNA analysis*.

*Multiple R^2^ = 0.413, adjusted R^2^ = 0.399, F = 29.56_df1 = 3, df2 = 126_, p = 1.54 × 10^−14^*.

Because there is multicollinearity in the model, partial Pearson's correlation coefficients were calculated to isolate the association between *bap1* and *ago2* expression from the effect of SINV RNA copies on either variable (Figure 4B). After accounting for the partial effect of log_10_ (SINV RNA copies) on *ago2* expression (*r* = 0.42, *p* = 1.87 × 10^−6^) *bap1* expression has a high partial correlation with *ago2* expression (*r* = 0.58, *p* = 8.42 × 10^−12^, Figure 4B). Therefore, *bap1* and *ago2* are likely co-regulated. It is intriguing to speculate that this co-expression could be due to functions in a common pathway. Alternatively, it could simply be case of co-expression for a similar function (i.e., antiviral).

## Conclusions

Honey bee antiviral defense mechanisms include the Toll and Imd (NFkB) signaling pathways and dsRNA-triggered mechanisms, including sequence-specific RNAi and a non-sequence specific triggered pathway that is less well-characterized (33, 36, 41, 44, 51, 115–119) [reviewed in (6, 120)]. Transcriptome level analysis and *in vivo* experiments determined that *bee antiviral protein-1* (*bap1*, GenBank: MF116383) plays an important role in honey bee antiviral defense, in the context of SINV-infection. Herein, we further characterized *bap1* using bioinformatic and phylogenetic approaches that revealed that *bap1* is taxonomically restricted to Hymenoptera and *B. germanica* and that it is evolving primarily under neutral selection, although some sites were under positive or negative selection. In line with those analyses, structural analysis predicted that Bap1 is highly disordered and neutral evolution is common for disordered proteins. We hypothesize that, like other disordered proteins, Bap1 may have the capability to bind many different protein or nucleic acid targets and thus serve as a molecular interaction hub in the honey bee antiviral defense. Future work will determine whether regions of positive selection are particularly important for interfacing with binding partners (i.e., nucleic acids or protein), and identifying those binding partners. To begin to identify the pathways *bap1* may interact with, a weighted gene correlation network analysis was performed and determined *bap1* expression is highly correlated with several immune genes, including *ago2* which was confirmed in an independent dataset. Whether coexpression of *bap1* and *ago2* is due to coregulation by a shared pathway or simply due to their shared antiviral function remains to be elucidated. Overall, these data indicated that *bap1* is an exciting avenue of research as a novel antiviral gene. Since host responses to specific viruses may vary, future studies that investigate the potential antiviral function of *bap1* in response to other viruses are required. The identification of new genes and pathways involved in combatting virus infections in honey bees may present opportunities to develop strategies that mitigate future honey bee colony deaths due to virus infection.

## Materials and Methods

### Age-Matched Live Honey Bees

Honey bee (*Apis mellifera)* colonies were established from packages (~1.5 kg of worker bees and a naturally-mated queen) of primarily *Apis mellifera carnica* stock purchased from a commercial producer in Montana in April 2018. Honey bees were kept in Langstroth hives located on Montana State University's Horticulture Farm in Bozeman, MT, US. Colonies were maintained using standard apicultural practices, including bi-monthly evaluation of *Varroa destructor* mite infestation levels using the powdered sugar roll method (121). Colonies were treated with formic acid polysaccharide gel strips (Mite Away Quick Strips®, Nature's Own Design Apiary Products) when mite infestation was >3% [3 mites per 100 bees (121)].

Honey bees for laboratory-based experiments were obtained from brood containing frames with newly emerging bees, which were collected 1 day prior to each experiment and maintained at 32°C and ~ 20% relative humidity in a laboratory incubator overnight. Young, age-matched (~24 h post-emergence), female adult bees were utilized for all experiments. For the duration of the experiment, honey bees were housed in modified deli-containers at 32°C and fed bee candy (powdered sugar mixed with corn syrup until pliable) and water *ad libitum* (33, 36, 47, 122).

### Double Stranded RNA Preparation

Double stranded RNA was generated by *in vitro* transcription with T7 RNA polymerase (22, 36). T7 promoter-containing PCR-products were amplified using primers listed in Supplementary Table S1, with the following thermocycler program: pre-incubation of 95°C (5 min), 35 cycles of 95°C (30 s), 60°C (30 s), and 72°C (1 min) followed by a final incubation at 72°C (5 min). PCR products were used as template for T7 polymerase transcription (100 μl reactions: NTPs (each 7.5 mM final), RNase OUT (40 units) (Invitrogen), buffer (400 mM HEPES pH 7.5, 120 mM MgCl_2_, 10 mM spermidine, 200 mM DTT); reactions were carried out at 37°C overnight (8–10 h). DNA was removed by adding 1 unit of RQ1 DNAse (Promega) and incubating for 15 min at 37°C. dsRNA products were ethanol precipitated with 1:10 volume 3M sodium acetate (pH 5.5), suspended in 200 μL RNase-free water, the RNA secondary structure was denatured *via* incubation at 100°C for 5 min, and then complementary RNA strands were annealed by slow cooling to room temperature. The dsRNA products were purified by phenol:chloroform extraction and subsequent ethanol precipitation with a 1:10 volume of 5M ammonium acetate. The precipitated dsRNAs were dissolved in 60–100 μL 10 mM Tris HCl (pH 7.5). The dsRNA quality was assessed by agarose gel electrophoresis and spectrophotometry. The dsRNA was quantified based on the band intensity relative to a standard with ImageJ version 1.50i (123).

### Virus Infection and Heat Shock Protocol

Honey bee virus infections were established *via* intra-thoracic injections using glass needles made by pulling borosilicate glass capillary tubes (100 mm long, 1 mL capacity, Kimble-Chase) with a coil temperature of 61°C using a PC-10 Dual-Stage Glass Micropipette Puller (Narishige). Prior to injection, age-matched honey bees (~24-h post-emergence) were cold anesthetized for 10 min at 4°C. Honey bees were infected with recombinant Sindbis virus expressing green fluorescent protein (SINV-GFP; 3,750 plaque forming units (pfu) in 2 μl 10 mM Tris HCl buffer pH 7.5) (33, 36, 124) *via* intra-thoracic injection using a Harbo syringe (Honey bee Insemination Service) and microcapillary glass needles; mock-infected bees were injected with 2 μL buffer (10 mM Tris HCl, pH 7.5).

Experimental treatment groups that were subjected to temperature stress were intrathoracically injected with either buffer or virus, allowed to recover for 6 h at 32°C, exposed to heat shock (i.e., 42°C for 4 h), and then transferred back to 32°C for the remainder of the study. The heat shock experiments were carried out using three independent honey bee cohorts obtained from three different colonies on distinct dates (i.e., replicate 1 in June 2018, replicate 2 in August 2018, and replicate 3 in July 2019). The data from these sampled are analyzed *post-hoc* from a previously published manuscript (47).

### RNA Extraction

Honey bee samples were dissected into head, thorax, and abdomen. The abdomen was chosen for further analysis as it is the primary site of immune cell generating fat bodies and it is distal from the site of injection, and thus virus infection naturally spread to that tissue. Honey bee abdomens were homogenized in 300 μL of deionized water with a sterile steel ball (5 mm) using a Tissue Lyser II (Qiagen) at 30 Hz for 2 min. Then 300 μL of TRIzol reagent (Invitrogen) was added to the homogenate, vortexed for 15 s and incubated at room temperature for 5 min. Next, 60 μL of chloroform was added, samples were shaken by hand for 15 s and incubated on the benchtop for another 2 min. Samples were then centrifuged at 12,000 × g at 4°C for 15 min and the aqueous phase was transferred to a clean centrifuge tube. One volume of isopropanol was added to the aqueous phase, mixed by inversion, and nucleic acid was precipitated by incubation at room temperature for 10 min. The precipitate was pelleted by centrifugation at 12,000 × g at 4°C for 10 min. Pellets were then washed with 500 μL of 75% ethanol and centrifuged at 7,500 × g at 4°C for 5 min, then air dried for 10 min at room temperature and dissolved in 30 μL of deionized water. RNA concentrations and quality were assessed on a Nanodrop 2000 spectrophotometer (Thermo Fisher). When quality was low, RNA was precipitated a second time by addition of four volumes of cold ethanol and 1:10 of a volume 3M sodium acetate (pH 5.5) and incubation at −20°C overnight. Nucleic acids were pelleted by centrifugation at 12,000 × g at 4°C for 10 min and pellets were washed one time with 500 μL 70% ethanol and centrifuged at 12,000 × g at 4°C for 5 min. Pellets were air dried and suspended in 30 μL dH_2_O. Samples were stored at −80°C until analysis.

### Reverse Transcription/cDNA Synthesis

Reverse transcription reactions were performed by incubating 2,000 ng total RNA, 200 units M-MLV reverse-transcriptase (Promega) and 500 ng random hexamer primers (IDT) for 1 h at 37°C, according to the manufacturer's instructions. cDNA was diluted 1:2 and 2 μL was used for PCR or qPCR analysis.

### Quantitative Polymerase Chain Reaction

Quantitative PCR (qPCR) was used to analyze the abundance of virus (i.e., SINV-GFP) and the relative abundances of honey bee immune gene and heat shock protein transcripts. All qPCR reactions were performed in triplicate with 2 μL of cDNA template. Each 20 μL reaction contained 1× ChoiceTaq Mastermix (Denville), 0.4 μM each forward and reverse primer, 1× SYBR Green (Life Technologies), and 3 mM MgCl_2_. A CFX Connect Real Time instrument (BioRad) was used for the following thermo-profile: pre-incubation 95°C for 1 min followed by 40 cycles of 95°C for 10 s, 58°C for 20 s, and 72°C for 15 s, with a final melt curve analysis at 65°C for 5 s to 95°C.

To quantify viral RNA copy numbers in the samples, SINV-GFP plasmid standards were used as templates, with concentrations ranging from 10^3^ to 10^9^ copies per reaction to create a linear standard curve. The detection limit was 10^3^ copies of the SINV cDNA using primers qSindbisFW4495 and qSindbisREV4635. The host gene *Am rpl8* was used as a reference gene to assess the relative expression of two immune genes (*Am bap1* and *Am ago2*). Amplicons were amplified in triplicate per sample for comparison, using primers in Supplementary Table S1. Reactions without template were carried out as negative controls. The qPCR specificity was verified through melt point analysis and *via* gel electrophoresis, and all products were previously verified by sequencing. The linear equation for the plasmid standard for SINV was: Ct = −3.348x + 40.25 (*R*^2^ = 0.996, efficiency = 98.9%) where “x” is the log (SINV genome equivalents). The relative expression of host genes was determined by a ranked ΔΔCt method in which the ΔCt was calculated by subtracting the *rpl8* Ct value from the Ct of the gene of interest. Then the ΔCt values were ranked to control for natural inter-individual variation in gene expression and the matching mock-infected ΔCt was subtracted from the treatment group ΔCt to obtain the ΔΔCt. The fold-change in cDNA abundance was calculated by the equation 2^−ΔΔCt^.

### Phylogenetic Analysis

*Bee antiviral protein-1* (*bap1*) (GenBank: MF116383) encodes an antiviral protein. The precise function of Bap1 has yet to be elucidated. Phylogenetic analyses of *bap1* were performed to gain functional insight. To perform a phylogenetic analysis, amino acid and nucleic acid sequences were acquired from NCBI non-redundant protein sequence database by searching using NCBI PSI BLAST function and exporting any sequence with an e-value below 0.001. An amino acid alignment was generated in MEGAX (57) using MUSCLE with default parameters.

A majority rule Bayesian consensus tree of Bap1 orthologs was derived from Bayesian analysis of amino acid alignment implemented in Mr. Bayes v3.2 using a Jones substitution model. Metropolis-coupled Markov Chain Monte Carlo (MCMC) permutation of parameters were initiated with a random tree and involved two runs each with 16 chains set at default temperatures (58, 59). Markov chains were run for 10,000,000 generations and sampled every 10,000 generations. MrBayes identified this sampling rate and a 25% burn-in as sufficient because of the non-autocorrelation of adjacently sampled trees and the complete convergence of the two separate MCMC runs at likelihood stationarity. Numbers on nodes are posterior probabilities (0-1), though posterior probabilities of 1 were not shown to improve figure clarity. The scale bar corresponds to proportion of amino acid change per site.

To perform PAML analysis, nucleic acid accession numbers were retrieved by creating a local BLAST database containing all of the taxa on the amino acid tree and performing BLAST analyses of all the amino acid sequences against the BLASTn database (54, 55). Nucleic acid sequences were retrieved by submitting the accession numbers to NCBI's batch ENTREZ (125). Sequences were again aligned by codon in MEGAX using MUSCLE with default parameters. Majority rule Bayesian consensus trees of *bap1* and *RPB1* orthologs were derived from a Bayesian analysis of a nucleic acid codon alignments implemented in Mr. Bayes v3.2 using the protein nucleic acid model. In this model, MrBayes first translates codons into protein and then infers relationships. Metropolis-coupled Markov Chain Monte Carlo (MCMC) permutation of parameters were initiated with a random tree and involved two runs each with 16 chains set at default temperatures (58, 59). Markov chains were run for 5,000,000 generations and sampled every 10,000 generations. MrBayes identified this sampling rate and a 25% burn-in as sufficient because of the non-autocorrelation of adjacently sampled trees and the complete convergence of the two separate MCMC runs at likelihood stationarity. Numbers on nodes are posterior probabilities (0-1), though posterior probabilities of 1 were left off to improve clarity. Generated trees were then partially edited in FigTree v 1.4.3 (126) and Adobe Illustrator to further improve clarity. The scale bar corresponds to proportional changes per codon site. Accession numbers are included on the branch tips and in Supplementary Tables S4, S5.

The codon alignments and the majority rule Bayesian consensus trees generated with this method were then used for selection analysis using the CODEML package in Phylogenetic Analysis by Maximum Likelihood (PAML) 4.9 (66). Relative rates of non-synonymous substitution (parameter ω) were estimated using fixed branch lengths for models M0, M1, and M2a. All models were run twice to check convergence. The log likelihood ratio test (LRT) statistic was calculated as twice the log likelihood difference between a test and null models (2Δ*lnL*) which was then compared against the χ^2^ distribution with critical values of 3.841 (M1-M0), 5.99 (M2-M1), and 7.814 (M2a-M0) at an α level of 0.05. PAML's Bayes Empirical Bayes (BEB) method was used on M2a to calculate the posterior probability that each amino site along Bap1 belonged to each site class (ω < 1, ω = 1, ω > 1). The dN/dS ratios calculated by the BEB method at each site containing an amino acid were then plotted as a stacked bar chart (Figure 2B; Supplementary Figure S2B). Site-specific dN/dS ratios were plotted and those with posterior probability of belonging to a given site class greater than an arbitrary cutoff of 0.8 were color coded as evolving under positive (red), neutral (blue) or negative (green) selection (Supplementary Figures S3, S4). Raw BEB probabilities and dN/dS ratios listed in Supplementary Tables S5, S8.

### Honey Bee RNAseq Data and Analysis

Honey bee transcriptome sequence data were generated and described in Brutscher et al. (36). Sequence data were deposited into NCBI Sequence Read Archive under accession number SRP101337 and is linked with NCBI BioProject #PRJNA377749. In brief, RNA samples obtained from individual honey bees (*n* = 47) from bees that were mock-infected, SINV-infected, dsRNA-treated, SINV + non-specific dsRNA, treated and SINV+ specific dsRNA treated. Samples were processed and the normalized number of Fragments Per Kilobase of transcript per Million mapped reads (FPKM) was determined using CuffDiff as described in Brutscher et al. (36, 127, 128) (Supplementary Table S10). FPKM values were log_2_ transformed and used for Weighted Gene Correlation Network Analysis (WGCNA).

### Weighted Gene Correlation Network Analysis

To identify genes are that co-expressed with *bap1* a weighted gene correlation network was constructed using the package WGCNA (86, 87) in R 4.0.2 from data analyzed *post-hoc* (36). WGCNA uses an unsupervised hierarchical clustering algorithm that groups genes into modules based on similarity of expression. In this case WGCNA analysis was performed on log_2_ transformed transcript FPKM values from three individuals in several treatment groups at several time points as described above. Recommended settings were used for the analysis (86, 87). However, briefly, minimum module size was set to 20 and deep split was set to 2. A signed correlation matrix was generated using default parameters, and a signed gene correlation network was constructed using a soft power threshold of 10. Modules were merged based on a branch cut height of 0.25. Trait data were used to identify module eigengenes differentially expressed in response to various traits (e.g., SINV RNA copies or whether or not the individual was injected with dsRNA) and plotted as a heat map (Supplementary Figure S5; Supplementary Tables S13–S20). The module that MF116383 belonged to was manually identified and pared down to include only the strongest connections with correlations above 0.5 or below −0.5. Then this network was imported into Cytoscape v 3.8.2 (129) for visualization and the *bap1* subnetwork was isolated manually (Supplementary Figure S6). For clarity, genes with no predicted function were removed from the network (Figure 3C). All remaining connections in this subnetwork are positive correlations above 0.5.

### Statistical Analysis

All statistical analyses were carried out in R 4.0.2, except the likelihood-ratio test statistics to compare models of selection which were calculated manually by subtracting the null model from the test model and multiplying by 2 (2ΔlnL). ANOVAs and linear models were constructed using the base R package (130, 131). Partial Pearson's Correlation coefficients were calculated using the *ppcor* package in R (132). Because our dataset contains zero values in our SINV measurement (untreated bees), SINV levels were log_10_ transformed prior to calculating correlation coefficients to improve homoscedasticity and improve normality.

## Data Availability Statement

Sequence data were deposited into NCBI Sequence Read Archive under Accession Number SRP101337 and is linked with NCBI BioProject PRJNA377749.

## Author Contributions

AJM and MLF: conceptualization, formal analysis, writing—original draft preparation, writing—review and editing, and visualization. AJM, KFD, and MLF: methodology. AJM, LMB, KFD, and MLF: investigation. MLF: resources, supervision, project administration, and funding acquisition. AJM, LMB, and MLF: data curation. All authors have read and agreed to the published version of the manuscript.

## Funding

The Flenniken laboratory was supported by the National Science Foundation CAREER Program (Award Number 1651561), Montana Department of Agriculture Specialty Crop Block Grant Program, United Department of Agriculture (USDA) - National Institute of Food and Agriculture (NIFA), Hatch Multistate Funding (NC-1173), and Project Apis m.-Costco Scholar Fellowship Program for Honey Bee Health. The funders had no role in study design, data collection and analysis, decision to publish, or preparation of this manuscript.

## Conflict of Interest

The authors declare that the research was conducted in the absence of any commercial or financial relationships that could be construed as a potential conflict of interest.

## Publisher's Note

All claims expressed in this article are solely those of the authors and do not necessarily represent those of their affiliated organizations, or those of the publisher, the editors and the reviewers. Any product that may be evaluated in this article, or claim that may be made by its manufacturer, is not guaranteed or endorsed by the publisher.
