# Supplementary material for: The Honey Bee Gene Bee Antiviral Protein-1 Is a Taxonomically Restricted Antiviral Immune Gene
Source: Front Insect Sci. 2021 Oct 20;1:749781. doi: 10.3389/finsc.2021.749781 (PMC10926557; doi:10.3389/finsc.2021.749781)
Supplement: Supplementary file 1 [file Data_Sheet_1.ZIP › Supplementary Figures_FINAL.pdf]

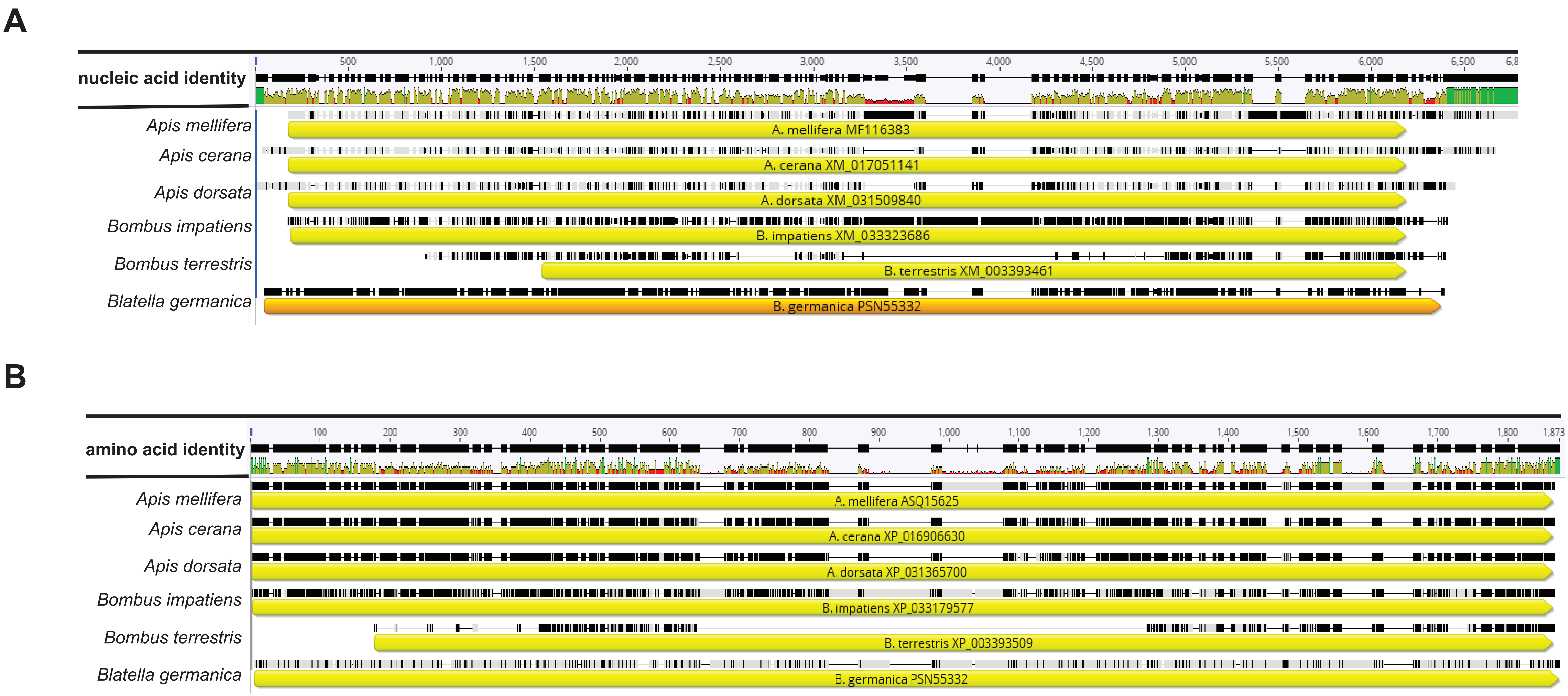

Supplemental Figure S1. Alignment of bap1 nucleic acid and amino acids sequences with several orthologues. Orthologues of bap1 were identified by PSI-BLAST and collected all sequences with a e-value below 0.001. A few select orthologues were chosen for visual representation here. Alignments were generated in Geneious using MUSCLE with default parameters. (A) Transcript nucleic acid sequences for bap1 (5,158 nt) orthologues from A. mellifera (MF116383), A. cerana (XM_ 017051141), A. dorsata (XM_031509840), B. impatiens (XM_033323686), B. terrestris (XM_003393461), and B. germanica (PSN55332). (B) Amino acid sequences for Bap1 (1,511 aa) orthologues from A mellifera (ASQ15625), A. cerana (XP_016906630), A. dorsata (XP_031365700), B. impatiens (XP_033179577), B. terrestris (XP_003393509) and B. germanica (PSN55332). See Supplementary table S3 for alignment pairwise identities.

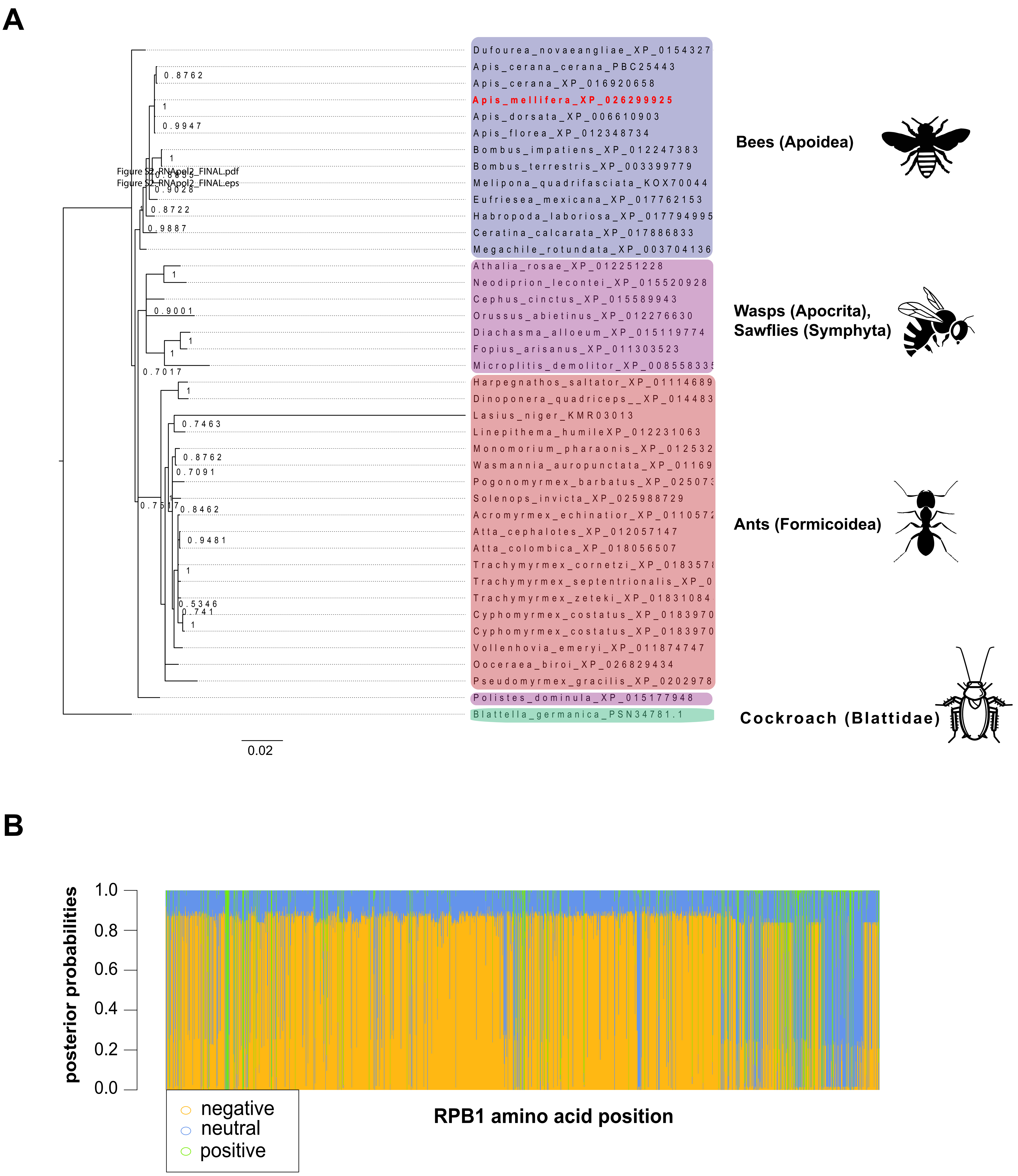

Supplemental Figure S2. RPB1 phylogenic relationship to other Hymenopteran orthologues inferred from amino acid sequence. (A) Majority rule Bayesian consensus tree of RPB1 homologues derived from Bayesian analysis of amino acid alignment implemented in Mr. Bayes v3.2 using a Jones substitution model. Numbers on branches and nodes are posterior probabilities (0-1), though posterior probabilities values of 1 are not shown to improve clarity. The scale bar corresponds to proportion of amino acid change. Accession numbers are included on the branch tips and in Supplemental Table S3.
(B) A corresponding majority Rule Bayesian consensus tree derived from Bayesian analysis of a codon alignment was used in a selection analysis in the CODEML package in Phylogenetic Analysis by Maximum Likelihood (PAML) 4.9. The Bayes Empirical Bayes method under model 2, which assumes 3 site classes (negative: < 1, neutral: = 1, and positive selection: > 1), was used to calculate the posterior probability that each amino acid position belonged to each site class. The posterior probabilities were then plotted as a stacked bar chart along the length of RPB1 amino acids. This method shows a significant proportion of RPB1 amino acids under negative selection.

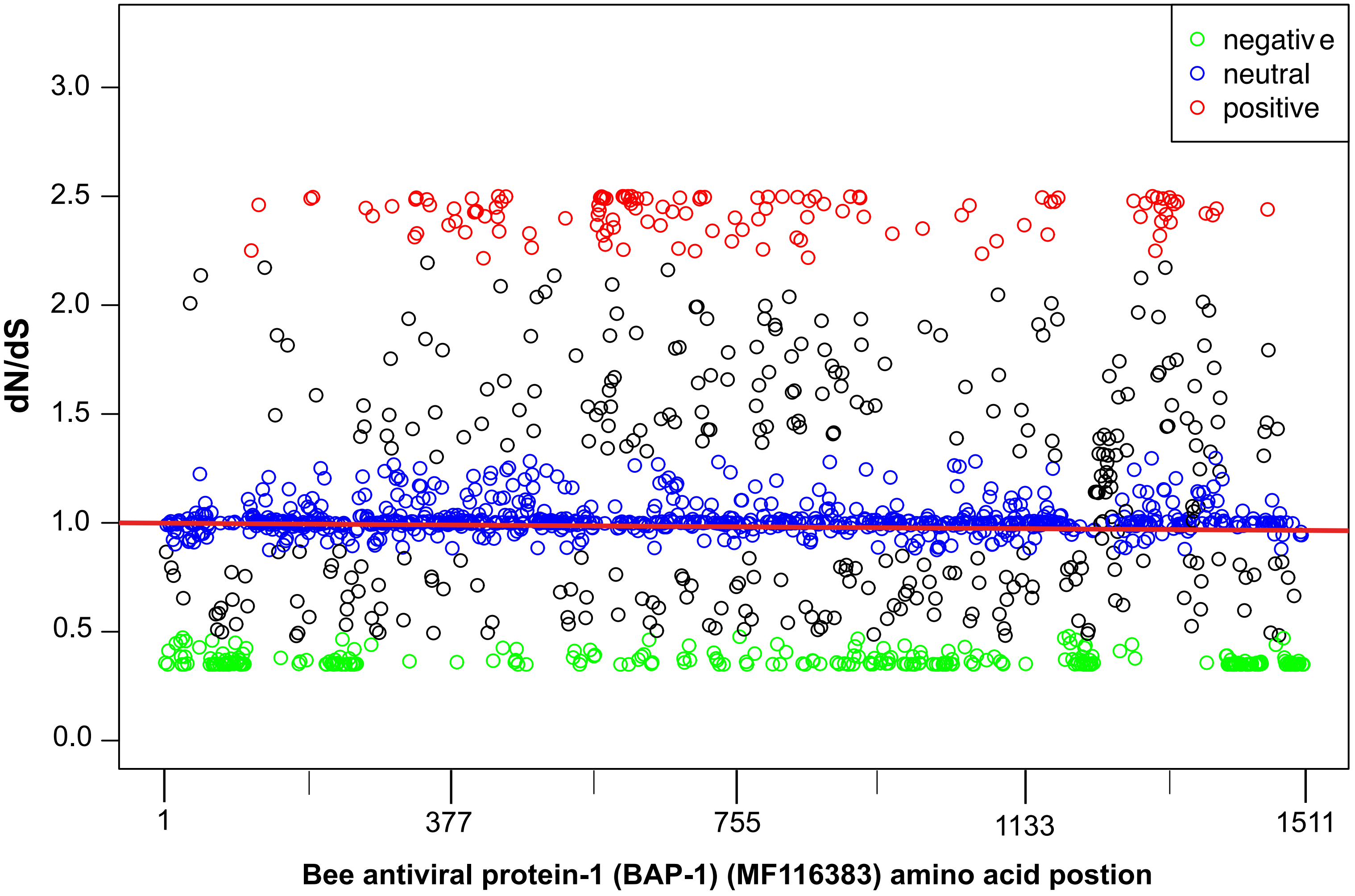

Supplemental Figure S3. Calculated dN/dS ratios at each bap1 amino acid position. The Bayes Empirical Bayes method in CODEML (PAML) was used to calculate dN/dS ratios and the posterior probabilities of being under negative, neutral, or positive evolution at each Bap1 amino acid position. Sites with a posterior probability of belonging to a particular site class larger than 0.8 were color coded as either green (negative, ω< 1), blue (neutral, ω = 1) or red (positive, ω > 1). A red line was placed at 1 to represent true neutral selection.

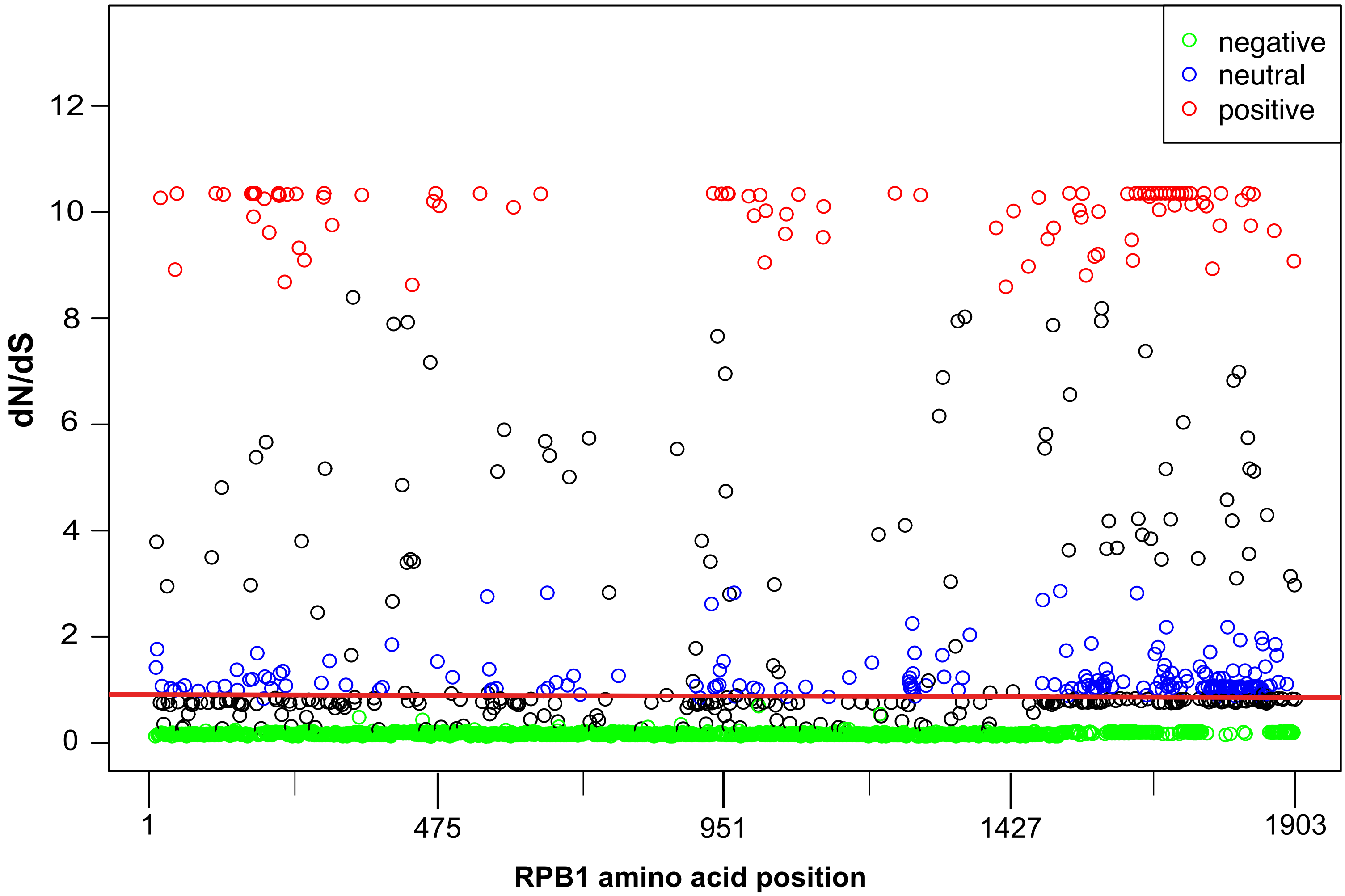

Supplemental Figure S4. Calculated dN/dS ratios at each RPB1 amino acid position. The Bayes Empirical Bayes method in CODEML (PAML) was used to calculate dN/dS ratios and the posterior probabilities of being under negative, neutral, or positive evolution at each RPB1 (the largest subunit of DNA-directed RNA polymerase II) amino acid position. Sites with a posterior probability of belonging to a particular site class larger than 0.8 were color coded as either green (negative, $\omega < 1$), blue (neutral, $\omega = 1$) or red (positive, $\omega > 1$). A red line was placed at 1 to represent true neutral selection.

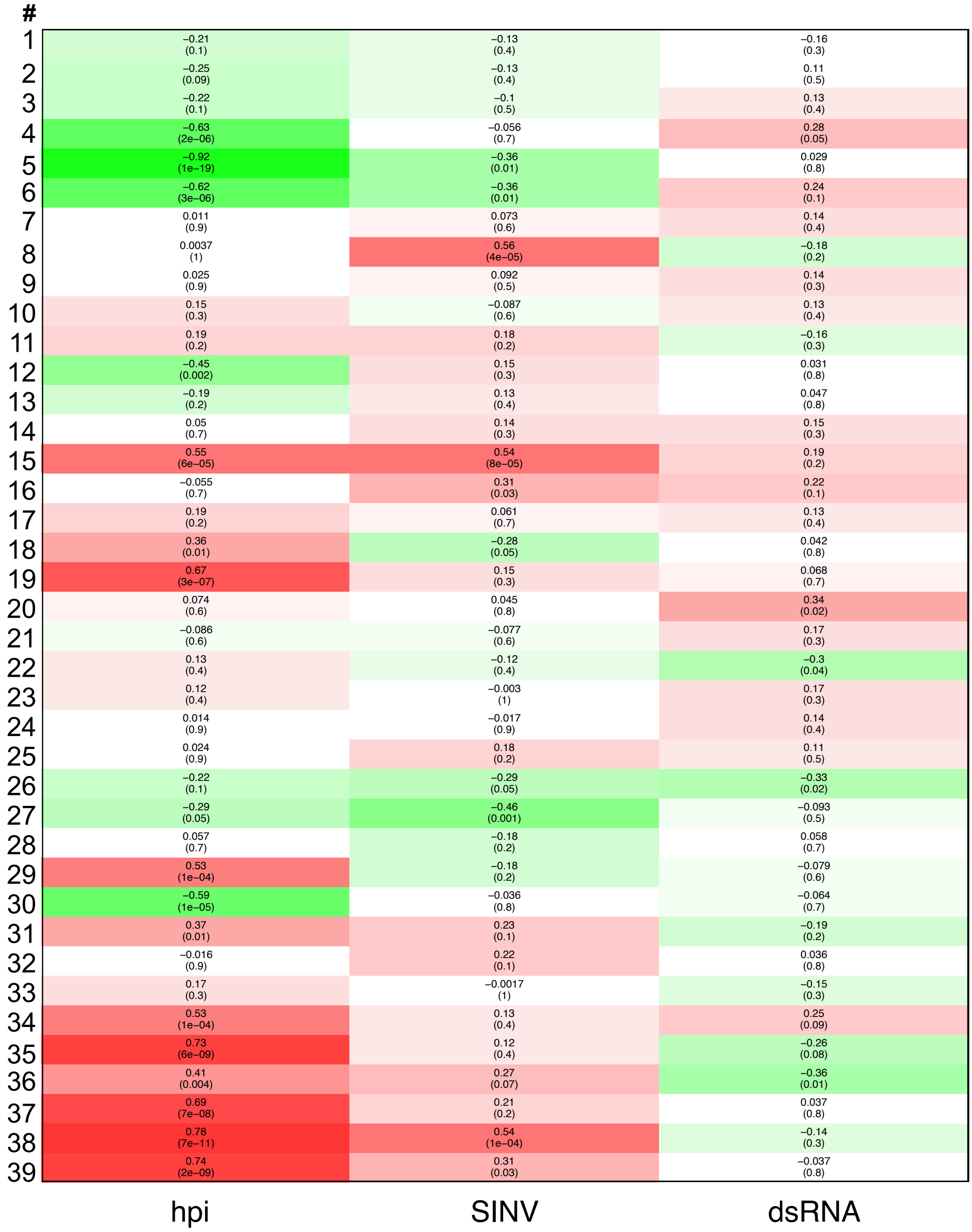

Supplemental Figure S5. Heatmap showing module-trait relationships. Weighted gene correlation network analysis (WGCNA) was used to identify 39 clusters of highly coexpressed genes (modules). The coexpression of each module was then correlated with additional traits (i.e., hours post infection, SINV levels, and whether or not the bee received dsRNA). The correlation coefficient for each module-trait pair is at the top of each box with the p-value in parentheses below it. Additionally, the darker the red, the more positive the correlation and the darker the green more negative the correlation. There were three gene modules that most correlated with virus (SINV) infection (i.e., #8, #15, and #38), and one module that best correlated with dsRNA treatment (i.e., #20). These modules were discussed further in the text. The trait values used to calculate these correlation coefficients are included in Supplemental Table S13. The gene-gene correlations of all modules that showed significant correlation with either SINV or dsRNA are included in Supplemental tables S14-21

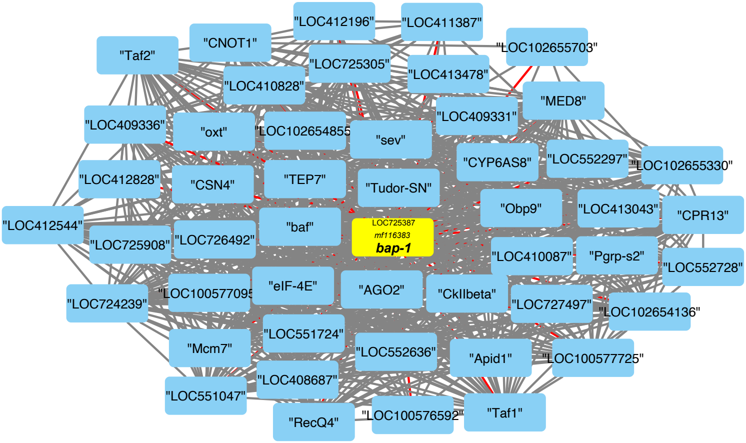

Supplemental Figure S6. Full bap1 coexpression subnetwork.
Weighted gene correlation network analysis (WGCNA) was used to identify 39 clusters of highly coexpressed genes (modules). Gene module #38 had a correlation coefficient of 0.54 (p = 1x10-4) with SINV RNA copies. This network was imported into Cytoscape v3.8.2 for visualization and boxes contain either the gene name or the NCBI locus designation. This module was also identified to contain bap1. In order to produce a more easily visualized network, only genes that shared edges with bap1 were retained. In addition to bap1 which is an antiviral protein, this subnetwork included several immune genes, most notably ago2, the core component of the RNAi induced silencing complex. Module #38 also contained TEP7 and TUDUR-SN, both proteins implicated in antiviral defense in insects.
